# Hot-rolling nanowire transparent electrodes for surface roughness minimization

**DOI:** 10.1186/1556-276X-9-310

**Published:** 2014-06-19

**Authors:** Hadi Hosseinzadeh Khaligh, Irene A Goldthorpe

**Affiliations:** 1 Department of Electrical and Computer Engineering, University of Waterloo, Waterloo, ON N2L 3G1, Canada; 2 Waterloo Institute for Nanotechnology, University of Waterloo, Waterloo, ON N2L 3G1, Canada

**Keywords:** Silver nanowires, Transparent electrode, Hot-rolling, Surface roughness, Adhesion, Organic electronics

## Abstract

Silver nanowire transparent electrodes are a promising alternative to transparent conductive oxides. However, their surface roughness presents a problem for their integration into devices with thin layers such as organic electronic devices. In this paper, hot rollers are used to soften plastic substrates with heat and mechanically press the nanowires into the substrate surface. By doing so, the root-mean-square surface roughness is reduced to 7 nm and the maximum peak-to-valley value is 30 nm, making the electrodes suitable for typical organic devices. This simple process requires no additional materials, which results in a higher transparency, and is compatible with roll-to-roll fabrication processes. In addition, the adhesion of the nanowires to the substrate significantly increases.

## Background

Transparent electrodes are a required component of many devices such as organic solar cells, electronic displays, and touch screens. The most commonly used transparent conductor is indium tin oxide (ITO). ITO, however, is expensive, not suitable for flexible applications, and requires sputtering, high temperatures, and vacuum for its deposition. Several materials have been proposed to replace ITO such as graphene [1], carbon nanotubes [2,3], and copper [4,5] and silver nanowires [6-8]. Of these, silver nanowire electrodes have been identified as the lead alternative because they have the lowest sheet resistance at a given transparency [9-11]. Not only can silver nanowire electrodes provide the same sheet resistance and transparency as ITO, but they are also highly flexible [12,13] and inexpensive [11], and their fabrication is compatible with roll-to-roll processes.

In spite of all the advantages of nanowire electrodes, there are certain issues that need to be addressed before their widespread use in devices. One of these most important issues is their surface roughness. Because there are typically junctions on an electrode where three or more nanowires are stacked on top of one another, maximum peak-to-valley values can reach three times the diameter of the nanowires or more [12,14]. Nanowires with diameters of 90 nm are commonly used, and so, these electrodes have peak-to-valley values around or exceeding 270 nm. This is problematic for many devices, especially ones that consist of thin layers. In organic electronic devices, for example, the low electron mobility and fast recombination times require organic layers to be less than 100-nm thick (typically 40 to 80 nm depending on the device and materials used) [15,16]. Several reports where silver nanowire electrodes have been used in organic solar cells have reported lower efficiencies than equivalent devices built on ITO. The rough surface of the nanowire electrodes causes a lower shunt resistance, which increases the dark current and hinders the efficiency of the solar cells [17-19]. The roughness also leads researchers to use a thicker layer of poly(3,4-ethylenedioxythiophene) poly(styrenesulfonate) (PEDOT:PSS) than is typically used on ITO-based devices [18]. This thicker layer decreases transparency and therefore also reduces efficiency. Weak adhesion of nanowires to the substrate is another important issue. Without any special processing, scratches or shear stresses on the surface can easily wipe the nanowires from the surface [11].

Several papers in the literature have addressed the roughness and adhesion issues of nanowire electrodes. Solutions fall into three general categories. The first involves using a transparent conductive material to fill the spaces between the nanowires [14,18,20-22]. Gaynor et al. pressed silver nanowires into a layer of the transparent conductive polymer (PEDOT:PSS) to decrease the root-mean-square (RMS) surface roughness to 12 nm and maximum peak-to-valley values to around 30 nm [21]. Choi et al. instead deposited the PEDOT:PSS layer on top of the nanowire film to achieve an RMS roughness of 52 nm [14]. Chung et al. alternatively used ITO nanoparticles to fill the spaces between the wires and reduced the RMS roughness to 13 nm and the maximum peak-to-valley to below 30 nm. In the latter paper, polyvinyl alcohol (PVA) was also added to the ITO nanoparticle solution to increase the adhesion of the nanoparticle/nanowire film to the substrate [22]. The downside of all these approaches is that to significantly reduce surface roughness, the required thickness of the conductive material needs to be at least three times the diameter of the nanowires. At these thicknesses, there is a reduction in the electrode transparency and consequently the efficiency of the devices due to the limited transparency of the conductive materials [18].

The second category to reduce roughness is to deposit a transparent but nonconductive polymer on top of the nanowire film [12,23-25]. This allows a material that is more transparent than PEDOT:PSS or ITO to be used. Using an optical adhesive in this manner, Miller et al. reduced the RMS roughness of silver nanowire films to 8 nm and there was only a 2% change in sheet resistance after an adhesion test [25]. Zeng et al. buried silver nanowires in PVA to reduce the surface RMS to below 5 nm and increase adhesion of the nanowires to the substrate [24]. However, because the polymers used are not conductive, in all these studies the nanowire/polymer composite must be peeled off the original substrate to expose the conductive nanowire-mesh surface, which adds a complex manufacturing step. Although not reported in the literature (to our knowledge), the nanowire film could instead be pressed into a transparent nonconductive polymer, to avoid the peeling step. This technique however would still be less than ideal as an extra polymer layer would still add manufacturing complexity and some devices may not be compatible with the polymer used.

The third category to reduce nanowire electrode roughness is to avoid using an additional polymer and instead press the nanowires into the plastic substrate itself (usually polyethylene terephthalate, PET) [7,26,27]. Tokuno et al. mechanically pressed silver nanowire films on PET at room temperature [26]. The resulting RMS surface roughness was 18 nm, which is still quite high. Hauger et al. added to this process by applying heat during pressing to soften the PET substrate [27]. In this latter paper, silver nanowire films on PET were placed facedown on a 165°C stainless steel sheet, and then a rod was rolled over the backside of the substrate. The resulting RMS surface roughness of the rolled electrodes was 27 nm, which is not as smooth as what other methods were able to achieve. After an adhesion test, which was done by applying and then peeling off a piece of scotch tape, the sheet resistance of the electrodes increased more than four times. Furthermore, the high temperature used is not compatible with most plastic substrates, and the maximum peak-to-valley values, which are more important than RMS values in regards to electrical shorts or shunting, were not reported.

This present study uses a roll-to-roll compatible process whereby hot rollers are used to apply heat and mechanical pressure at the same time. The heat results in the softening of the plastic substrate while the mechanical pressure pushes the silver nanowires into the surface of the softened substrate. By embedding the silver nanowires into the substrate surface, the RMS roughness is reduced to 7 nm and the maximum peak-to-valley is 30 nm. A temperature of 80°C was used, which is safe for most plastic substrates. No additional polymers are used which results in higher transparencies, reduces the number of manufacturing steps, and avoids potential incompatibilities between extraneous polymers and some device materials.

## Methods

### Fabrication of electrodes

**Figure 1:**
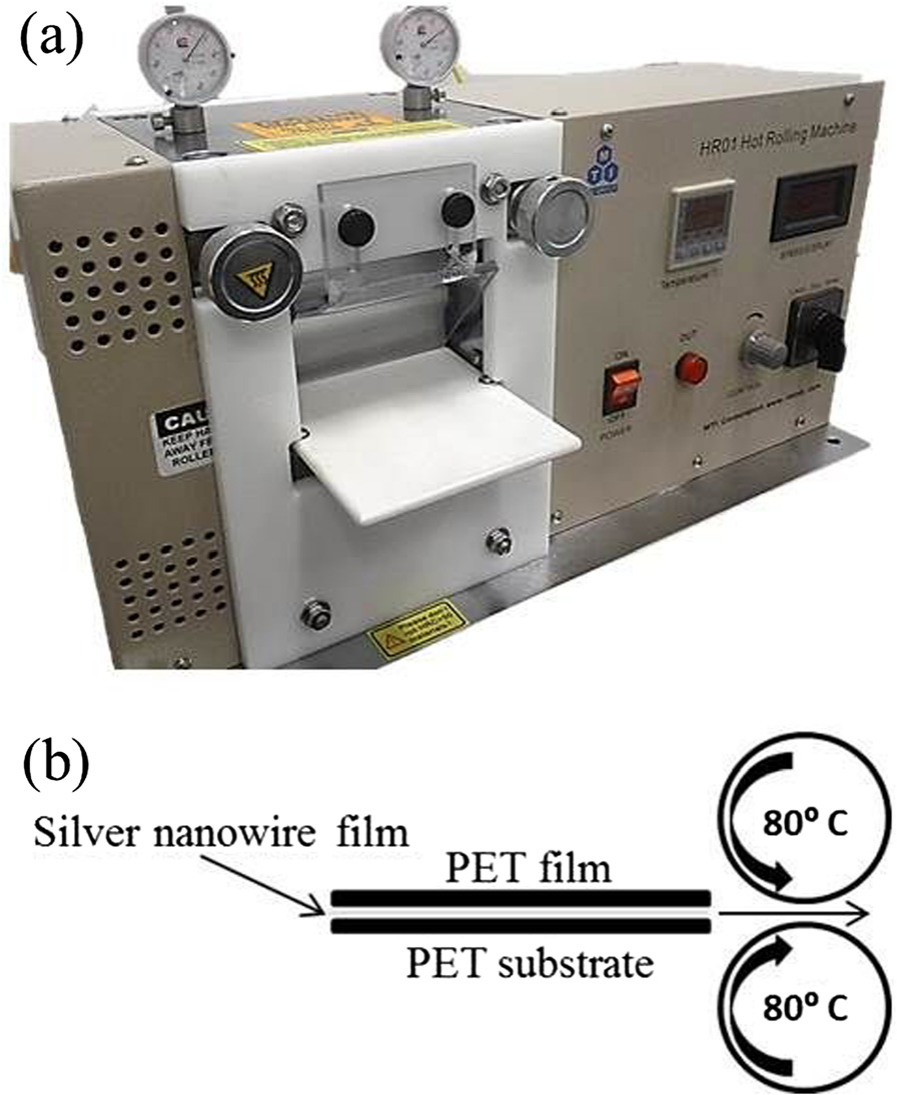
**Rolling process of the nanowire electrodes. (a)** The hot-rolling press. **(b)** Schematic of the rolling process.

Silver nanowires dispersed in ethanol were purchased from Blue Nano Inc., Charlotte, NC, USA, with an average diameter of 35 nm and an average length of 15 μm. Heat stabilized PET film with a thickness of 127 μm was purchased from Dupont Tianjin Inc., Tianjin, China. The PET film had an RMS roughness of 2 nm. Films of silver nanowires were deposited uniformly on 5 cm × 5 cm PET substrates using the Mayer rod coating technique [2,7,8] and then rinsed with acetone to remove the polyvinylpyrrolidone (PVP) layer on the nanowire surfaces which was left over from the nanowire synthesis process. Pressing was done with a hot-rolling press (MSK-HRP-01, MTI Corporation, Richmond, USA Figure 1a). The electrodes were first rolled two times at room temperature so that the nanowires adhered to the PET. The rolling speed was 5 mm/s and the spacing between the two rollers was 60 μm. The temperature of the rollers was then raised to 80°C and the electrodes were rolled two more times. Because the surfaces of the metal rollers are relatively rough, this leads to an uneven pressure which can deform the substrate and damage the nanowires. Therefore, during the hot rolling, a smooth plain PET film was placed between the silver nanowire film and the top roller. The nanowires do not stick to this top PET film because of the initial room temperature rolling step. Figure 1b shows the schematic of the hot-rolling process. As reference samples, some electrodes were not pressed but instead annealed in a furnace at 100°C for 30 min, which is a common way of preparing silver nanowire electrodes [7,19].

### Characterization

The sheet resistance of the electrodes was measured by either a four-point probe measurement or a multimeter. The transparencies were recorded with a spectrophotometer, with plain PET as a reference. Atomic force microscopy (AFM) was used to measure surface roughness, and peak-to-valley values were extracted from line scan data collected by Gwiddion software. Tilted scanning electron microscopy (SEM) images were taken of the electrodes, which had been coated with a 10-nm gold layer to prevent electron charging. To determine the level of adhesion, a piece of scotch tape was applied on the silver nanowire film, pressed with a finger, and then peeled off, with the sheet resistance of the electrode being measured before and after. Bending tests were done by bending the electrodes around a rod with a 5-mm radius. The sheet resistance of the electrodes was measured before, after, and during the bending.

## Results and discussion

The rollers’ temperature, speed, and spacing were optimized to minimize the surface roughness of the electrode without damaging the silver nanowires and the substrate. A rolling temperature of 80°C was the maximum that the substrate could tolerate before deforming. The rolling speed of 5 mm/s allowed enough time for the substrate to heat up and soften during rolling.

**Figure 2:**
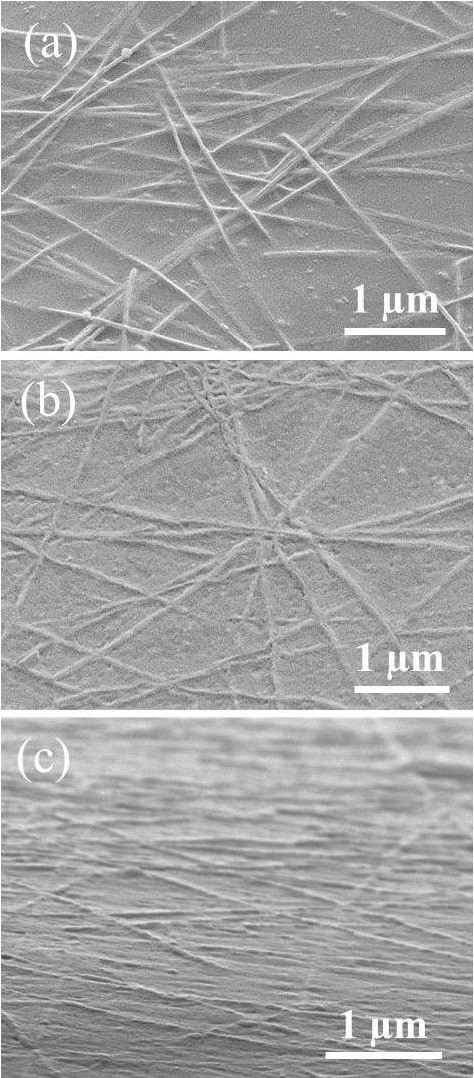
**Images of the nanowire electrodes.** SEM images of tilted (45°) silver nanowire films on PET after **(a)** annealing and **(b)** hot rolling. **(c)** SEM image of a tilted (85°) hot-rolled electrode, which shows that the nanowires are embedded in the substrate surface.

Figure 2 shows SEM images of an unpressed, annealed reference sample and a hot-rolled electrode. It can be seen that the hot-rolled nanowires are pushed into the substrate with the nanowires remaining at the surface so that they can contact a device layer above it. The annealed electrode had a sheet resistance of 22 Ω/sq with a specular transparency of 93% at 550 nm, while the hot-rolled electrode with the same density of nanowires had a sheet resistance of 14 Ω/sq, with 91% transmittance. Figure 2 indicates that hot rolling welds overlapping wires, which lowers the resistance of the nanowire junctions and explains the 35% lower sheet resistance of the hot-rolled electrodes. In contrast, the junctions on the annealed sample are not completely welded; an annealing temperature higher than 100°C cannot be used because of the plastic substrate. The transparency of the hot-rolled electrode was slightly lower than that of the annealed one, which may be due to a slight flattening of the nanowires. The sheet resistance and transparency of the hot-rolled electrodes match those of ITO electrodes on glass substrates [28] and are superior than ITO-coated plastic [29-31].

**Figure 3:**
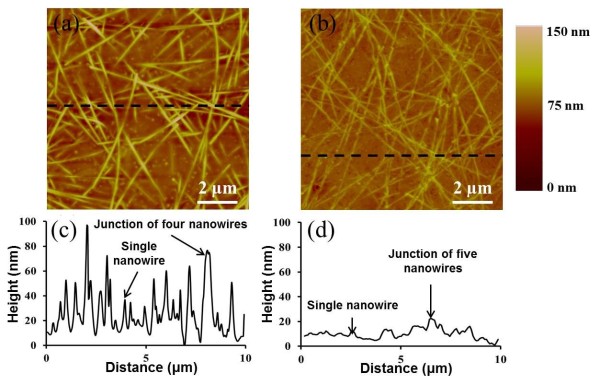
**Topography of the hot-rolled electrodes.** AFM images of silver nanowire electrodes on PET after **(a)** annealing and **(b)** hot-rolling. **(c)**, **(d)** Line scan data corresponding to the black dashed lines in **(a)** and **(b)**, respectively.

Figure 3 shows the AFM images of an annealed electrode and a hot-rolled electrode, with representative line scans underneath. Table 1 summarizes the RMS surface roughness and maximum peak-to-valley data for the annealed and hot-rolled electrodes. The surface roughness of the hot-rolled electrodes, measured over three similar samples, dropped 50% compared to that of the annealed sample to 7 nm, and the maximum peak-to-valley height was reduced to less than 30 nm. These roughness values are the lowest among electrodes which do not use additional materials to fill the spaces between the nanowires, and comparable to those that do. Furthermore, for a given sheet resistance, the hot-rolled electrodes are more transparent than electrodes that use additional materials [12,21]. The maximum peak-to-valley value of the hot-rolled electrodes is lower than the typical layer thicknesses in organic electronic devices.

**Table 1:** Roughness data of the nanowire electrodes

	**RMS roughness (nm)**	**Max peak-to-valley (nm)**
**Annealed**	14	>90
**Rolled at 80°C**	7	<30

Because different groups use different nanowire diameters for their electrodes, samples were also fabricated from 90-nm-diameter silver nanowires for comparison. The RMS roughness of the annealed 90-nm-diameter nanowire electrodes was 40 nm, and was 10 nm in the hot-rolled samples. The maximum peak-to-valley height values were 150 and 50 nm for the annealed and hot-rolled electrodes, respectively.

The results of the scotch tape test are tabulated in Table 2. The data indicate that, unlike as-deposited and annealed substrates, the nanowires in the hot-rolled electrode adhere to the substrate very well. The sheet resistance of the hot-rolled electrode was 14.0 and 14.1 Ω/sq before and after applying and removing the tape. This level of nanowire adhesion greatly exceeds other nanowire electrodes that were mechanically pressed [7,27].

**Table 2:** Percent change in sheet resistance after the tape test on differently prepared electrodes

	**As-deposited**	**Annealed**	**Rolled at 80°C**
Sheet resistance change after tape test	Open circuit	510%	0.9%

While bent around a 5-mm rod, the sheet resistance of hot-rolled electrodes increased by less than 1%. When bent 100 times and then returned flat, the resistance was unchanged. In comparison, the sheet resistance of annealed electrodes increased by 3% when bent, and 2% after 100 bending cycles. Thus, the hot-rolled electrodes had slightly better flexibility, perhaps due to the better attachment of the nanowires to the substrate.

## Conclusion

This paper demonstrates a hot-rolling process to achieve silver nanowire transparent electrodes with a smooth surface topology and excellent nanowire adhesion to the substrate. An RMS surface roughness of 7 nm was achieved, with a maximum peak-to-valley height of 30 nm. These values meet the smoothness requirements needed for most organic devices. The silver nanowires were successfully embedded in the substrate such that their sheet resistance changed less than 1% after the tape test. This report shows that the surface roughness issue for nanowire electrodes can be easily addressed in a roll-to-roll compatible process without using any additional materials.

## Abbreviations

AFM: atomic force microscopy; ITO: indium tin oxide; PEDOT:PSS: poly(3,4-ethylenedioxythiophene) poly(styrenesulfonate); PET: polyethylene terephthalate; PVA: polyvinyl alcohol; RMS: root-mean-square; SEM: scanning electron microscope.

## Competing interests

The authors declare that they have no competing interests.

## Authors’ contributions

HHK participated in the design of the study, carried out the experiments, and drafted the manuscript. IAG supervised the project, participated in the design of the study and analysis of its results, and revised the manuscript. Both authors read and approved the final manuscript.
